# The role of correspondence analysis in medical research

**DOI:** 10.3389/fpubh.2024.1362699

**Published:** 2024-03-22

**Authors:** Bojan Žlahtič, Peter Kokol, Helena Blažun Vošner, Jernej Završnik

**Affiliations:** ^1^Faculty of Electrical Engineering and Computer Science, University of Maribor, Maribor, Slovenia; ^2^Community Healthcare Center dr. Adolf Drolc, Maribor, Slovenia; ^3^Faculty of Health and Social Sciences Slovenj Gradec, Slovenj Gradec, Slovenia; ^4^Alma Mater Europaea, Maribor, Slovenia

**Keywords:** public health, medical research, correspondence analysis, exploratory data analysis, bibliometrics

## Abstract

Correspondence analysis (CA) is a multivariate statistical and visualization technique. CA is extremely useful in analyzing either two- or multi-way contingency tables, representing some degree of correspondence between columns and rows. The CA results are visualized in easy-to-interpret “bi–plots,” where the proximity of items (values of categorical variables) represents the degree of association between presented items. In other words, items positioned near each other are more associated than those located farther away. Each bi-plot has two dimensions, named during the analysis. The naming of dimensions adds a qualitative aspect to the analysis. Correspondence analysis may support medical professionals in finding answers to many important questions related to health, wellbeing, quality of life, and similar topics in a simpler but more informal way than by using more complex statistical or machine learning approaches. In that way, it can be used for dimension reduction and data simplification, clustering, classification, feature selection, knowledge extraction, visualization of adverse effects, or pattern detection.

## 1 Introduction

This “perspective article” aims to demonstrate the usefulness of correspondence analysis (CA) and inform the medical community about possible CA benefits in research and everyday practice settings. Correspondence analysis is a multivariate statistical and visualization technique. When a contingency table consists of two variables, we talk about a simple CA; however, if the analysis is extended to more than two categorical variables, it is called a multiple CA (MCA). The roots of correspondence analysis date back to 1935 when Herman Otto Hartly (born Hirschfeld) published his work on contingency tables (1). Based on Hartly’s work, Benzecri developed CA’s mathematical foundations during the 1960s in France (2). However, the method became popular outside France through the work of Michael Greenacre (2) and Leabrt and coworkers (3). Greenacre and coworkers also popularized the use of CA in medical research (4). However, some attempts to use CA in healthcare have been made since 1975, mainly by French authors (5–9).

Variants of CA and MCA are extremely useful in analyzing either two- or multi-way contingency tables, representing some degree of correspondence between two or more categorical variables. They translate deviations from the independence model in a contingency table into distances. Conceptually, they are similar to principal component analysis but apply to categorical rather than continuous variables. CA and MCA enable users to graphically display row and column categories and visually inspect their associations. There are several extensions of CA and MCA (10), such as constrained, aggregate, or canonical correspondence analysis (5).

The CA results are presented in so-called “bi–plots,” where the proximity of items (values of categorical variables) represents the degree of association between presented items. In other words, items positioned near each other are more associated than those located farther away. Each bi-plot has two dimensions, named during the analysis. The naming of dimensions adds a qualitative aspect to the analysis (6). It is worth noting that besides bi-plots, other graphical outputs, such as dendrograms or similarity trees, are commonly used when CA is employed for quali-quantitative analysis (7).

## 2 General benefits of correspondence analysis

The main benefits of CA are as follows:

- It shows relations and their strength between categorical categories in a way anyone can easily understand.
- It is objective because there are no underlying statistical distributional assumptions.
- It can be used on all types of categorical variables.
- It is a multivariate method.
- It provides a simple visualization of data.

## 3 Correspondence analysis in medical research and practice

To analyze the scope of CA use in medicine, we retrieved the corpus of publication from the Scopus indexing database (Elsevier, Netherlands). Scopus was chosen because it is considered reliable and authoritative and is the largest abstract and citation database of the research literature, including almost 50,000 source titles from more than 12,500 publishers. Scopus also covers MEDLINE and EMBASE databases and most of the Web of Science content. In addition, it provides advanced analytics services and enables 20,000 records to be exported simultaneously. The search was performed applying the following search string: {Correspondence analysis} in publication titles, abstracts, and keywords. The search was limited to the subject area of *Medicine.* The use of Curly Brackets {} denotes an exact search. In that way, we harvested 1,939 publications used in further bibliometric-based analysis (8).

**Figure 1:**
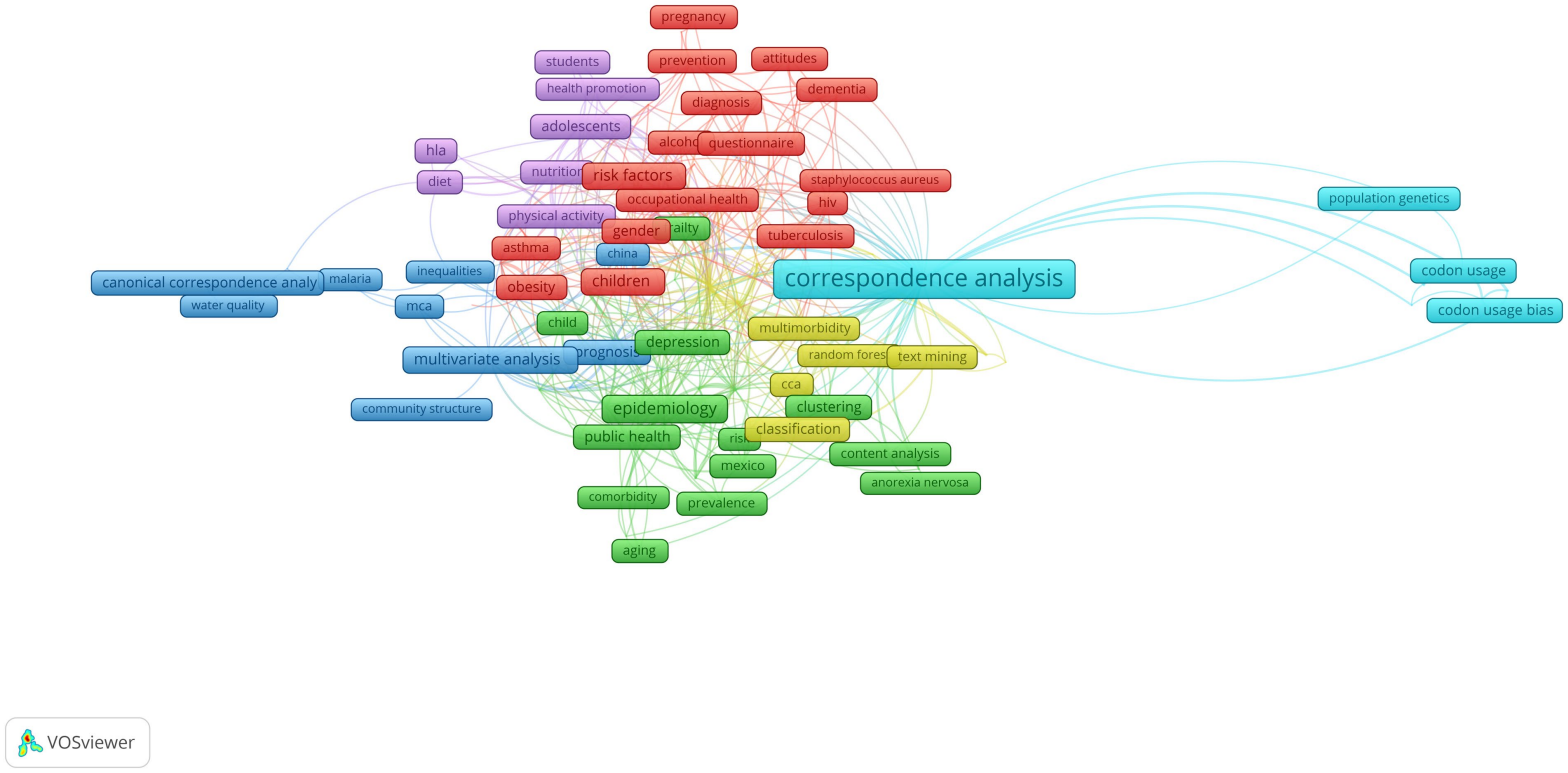
The research landscape of the role of correspondence analysis in medical research and practice.

The number of publications increased from 9 published in the year 1990 to 20 published in the year 2000, 63 in the year 2010, and 156 in the year 2022. The corpus of publications was analyzed using synthetic knowledge synthesis, a triangulation of bibliometrics, bibliometric mapping, and content analysis (11). For this perspective study, bibliometric mapping was performed using VOSViewer software, version 1.6.20 (9); however, other mapping software, such as Bibliometrix (12), exist, which could be used similarly. Bibliometric mapping on authors’ keywords resulted in four clusters, represented by different colors, as shown in Figure 1. By applying content analysis to cluster terms, we identified four themes, each presenting the use of correspondence analysis in Medicine. The themes, together with influential and interesting studies, are explained below:

- *Correspondence analysis in genetics (light blue cluster):* Correspondence analysis was used to investigate the relationship between transcriptional programs of the osteoarthritis genetic landscape and clinical outcomes using the severity index (13). In a retrospective study regarding clinical pathological characteristics and outcomes of triple-negative breast cancers, correspondence analysis was used in the investigation of the relationship between androgen receptor protein expression, core-needle biopsy (using different cutoffs), and standard clinical and pathological variables, including stromal tumor-infiltrating lymphocytes (14).
- *Multiple correspondence analysis combined with machine learning (yellow cluster):* Multiple correspondence analysis and random forests were used to analyze the linkage among socio-demographic, behavioral, psycho-social, and biological factors associated with high HIV RNA viral load (15). Data from the first wave of COVID-19 in New Zealand were analyzed comprising PCR-confirmed and symptomatic PCR-negative individuals using multiple correspondence analysis in combination with various machine learning algorithms (11).
- *Epidemiology and public health (green and blue cluster):* Responses to the questionnaire regarding policies, guidelines, civil awareness, epidemiology and data, detection rate, and care management of 102 countries were analyzed using multiple correspondence analysis to asses the country preparedness for management of non-alcoholic fatty liver disease (16). An online survey administered to the Italian population was analyzed using multiple correspondence analysis to detect the factorial dimensions underpinning ways of interpreting the social environment regarding decision-making, people’s mindsets, and similar aspects during the COVID-19 pandemic (17).
- *Healthy and active living (violet cluster):* Multiple correspondence analysis was used to classify youths into mental health profiles. Adolescents were categorized into three mental health profiles based on their mental wellbeing, resilience, quality of life, cognitive and behavioral disorder symptoms, and use of tobacco, alcohol, and similar substances (18). Multiple correspondence analysis combined with clustering was used to explore potential changes in dietary intake, physical activity, body weight, and food supply relative to individual characteristics during the COVID-19 lockdown (19).
- *Multiple correspondence analysis in primary healthcare (red cluster):* Multiple correspondence analysis was used to analyze the association of physical health multimorbidity in patients with and without severe mental illness (20). Multiple correspondence analysis was also applied to classify asthma into six subtypes based on data gathered from a large number of longitudinal primary care electronic health records (21).

## 4 Dissuasion: benefits of using correspondence analysis in medical research

In addition to the general benefits of using correspondence analysis addressed above, healthcare professionals with just a short training in CA can use it for complex tasks such as:

- dimension reduction and data simplification
- clustering
- classification
- feature selection
- knowledge extraction
- qualitative component
- visualization of adverse effect
- pattern detection

**Figure 2:**
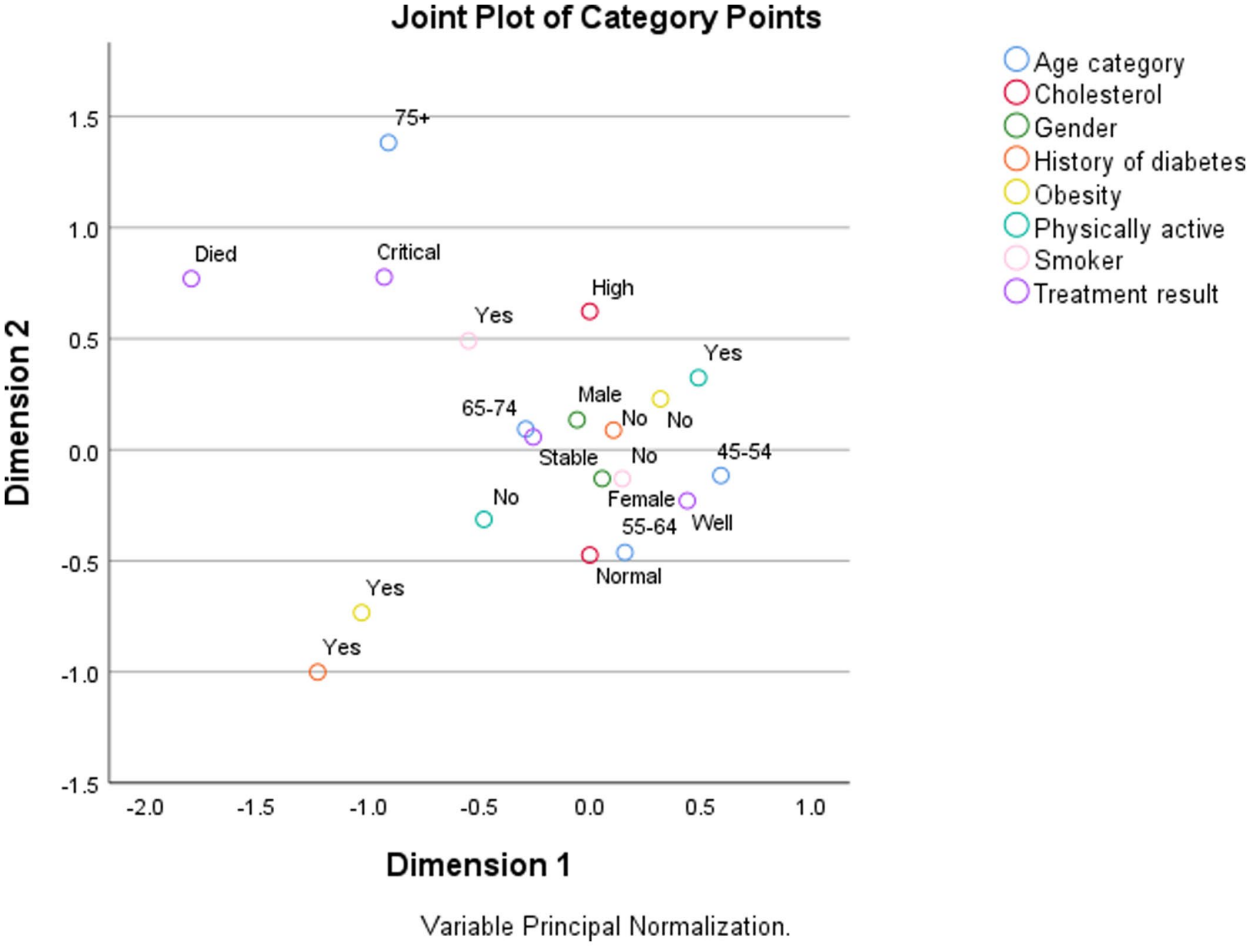
An example of using correspondence analysis in medical research.

Figure 2 demonstrates the results of the use of multiple correspondence analysis on the STROKE database. The STROKE database is provided by SPSS (IBM, Rochester, United States) in its sample set. It contains cleansed medical data for approximately 2,412 stroke patients collected from 20 hospitals. The data consist of demographic variables such as sex, age, physical activity, smoking status, health status variables including the presence of obesity, high cholesterol, and diabetes, and finally, the variable presenting the treatment outcome. The analysis was performed with SPSS, Version 29 (IBM, Rochester, United States). Figure 2 reveals that physically active women younger than 64 years with normal cholesterol are located near the treatment result “Well” category. On the other hand, patients older than 75 years and smoking are located near the treatment result “Critical” category. This evidence can be used for classification or clustering. The same evidence shows that age, smoking, cholesterol, and physical activity are important variables (Feature selection). On the other hand, the values of obesity and history of diabetes are located far from the treatment result in the “Critical or Death” category, and the male and female sexes are located very near each other, which might indicate that those variables are not so crucial for stroke management (dimension reduction and data simplification). The above evidence also contributes to pattern recognition and knowledge extraction. Dimension 1 could be labeled age and treatment results, which could qualitatively mean that the treatment results after stroke will worsen with aging. Dimension 2 could be labeled high cholesterol and smoking, qualitatively meaning that both have considerable adverse effects on stroke management.

## 5 Conclusion

The use of correspondence analysis in medicine is growing exponentially. As revealed in our study, it is employed in an increased number of different medical contexts for various tasks. Based on our findings, we believe that correspondence analysis may support medical professionals in finding answers to many important questions related to health, wellbeing, quality of life, and similar topics in a more straightforward but informal way than by using more complex statistical or machine learning approaches.

## Data availability statement

The original contributions presented in the study are included in the article/supplementary material, further inquiries can be directed to the corresponding author.

## Author contributions

BŽ: Conceptualization, Data curation, Methodology, Writing – original draft, Writing – review & editing. PK: Conceptualization, Data curation, Methodology, Visualization, Writing – original draft, Writing – review & editing. HB: Supervision, Validation, Writing – original draft, Writing – review & editing. JZ: Supervision, Validation, Writing – original draft, Writing – review & editing.
